# Transforming the Effectiveness and Equity of a Psychological Therapy Service: A Case Study in the English NHS Talking Therapies Program

**DOI:** 10.1007/s10488-024-01403-0

**Published:** 2024-08-17

**Authors:** Katy James, David Saxon, Michael Barkham

**Affiliations:** 1 Department of Psychology, University of Sheffield, Norfolk and Suffolk NHS Foundation Trust, Vita Health Group, Sheffield, England; 2 Clinical and Applied Psychology Unit, Department of Psychology, University of Sheffield, 1 Vicar Lane, Sheffield, S1 2LT England

**Keywords:** Patient outcomes, Therapist effects, Feedback, Learning health system, Effectiveness, Equity

## Abstract

To work with a psychological therapies service to implement a recovery plan, as required by a government body, aimed at improving patient outcomes (effectiveness) and decreasing practitioner variability (equity). A case-study utilizing components of a learning health system, including nationally mandated patient outcome data, comprising three 18-month phases: (1) retrospective baseline; (2) improving patient outcomes (management-led); and (3) reducing practitioner variability (clinician-led). Primary analyses focused on 35 practitioners (N_PR_ = 35) who were constant across the three phases and their patients in each phase (N_PA_ = 930, 1226, 1217, respectively). Reliable improvement rates determined patient outcomes and multilevel modeling yielded practitioner effects. To test generalizability, results were compared to the whole practitioner sample for each phase: (1) N_PR_ = 81, N_PA_ = 1982; (2) N_PR_ = 80, N_PA_ = 2227; (3) N_PR_ = 74, N_PA_ = 2267. Ethical approval was granted by the Health Research Authority. Patient outcomes improved in successive phases for both the core and whole practitioner samples with the largest impact occurring in the management-led intervention. Practitioner variability decreased in successive phases in both the core and whole practitioner samples except in the management-led intervention of the whole sample. Compared with the management-led intervention, the practitioner-led intervention yielded a decrease in practitioner effect exceeding 60% in the core sample and approaching 50% in the whole sample. The implementation of multiple components of a learning health system can lead to improvements in both the effectiveness and equity of a psychological therapy service.

**Supplementary Information:**

The online version contains supplementary material available at 10.1007/s10488-024-01403-0.

## Clinical or Methodological Significance of this Article

A sequential combination of management-led interventions targeted at improving patient outcomes followed by a compassionate and bespoke feedback to practitioners utilizing principles derived from deliberate practice is a feasible strategy for improving patient outcomes (effectiveness) and reducing practitioner variability (equity), thereby yielding a more equitable delivery of patient care.

**Supplementary Information:**

The online version contains supplementary material available at 10.1007/s10488-024-01403-0.

While a majority of research in the psychological therapies has focused on the relative efficacy and effectiveness of differing treatment modalities (e.g., Barkham & Lambert, 2021), there has been a gradual increase in research focused on the contribution of therapists, their intrinsic variability, and their effects on treatment outcomes. The study of therapists and their effects was noted in the classic study by Ricks (1974), followed by key contributions from Martindale (1978), Crits-Cristoph & Mintz (1991), and culminating in substantive reviews in successive 6th and 7th editions of *Bergin and Garfield’s Handbook of Psychotherapy and Behavior Change* (Baldwin & Imel., 2013; Wampold & Owen, 2021). Therapist effects have been consistently reported as being in the region of 5–7%, with the majority of studies deriving from practice-based (i.e., observational) studies (Baldwin & Imel, 2013; Johns et al., 2017). While the yield from randomized controlled trials of treatment effects are invariably assigned greater value than those derived from observational data, therapist selection and therapist sample size in any trial are likely to produce estimates of therapist effects that are either attenuated or unreliable. By contrast, therapist variability is a naturally-occurring phenomenon in practice-based research and, as such, introduces a level of inequity such that some patients are likely to be assigned to therapists who are either consistently more or less effective than others in terms of their patient outcomes.

In one of the largest studies of therapist effects to-date, Saxon and Barkham (2012) reported a therapist effect of 6.6%. Interestingly, the authors calculated that removal of the 19 therapists who were consistently yielding below average outcomes for their patients would result in a reduction by 2% in the therapist effect to 4.6% while the patient recovery rate would improve from 61.6 to 64.9%. Alternatively, if those same patients were treated by the average effective therapist, then an additional 265 patients would have recovered. Hence, there are real-world consequences in terms of patient outcomes as a result of significant therapist variability. However, few studies to date have investigated whether it is possible to reduce therapist variability.

**Fig. 1:**
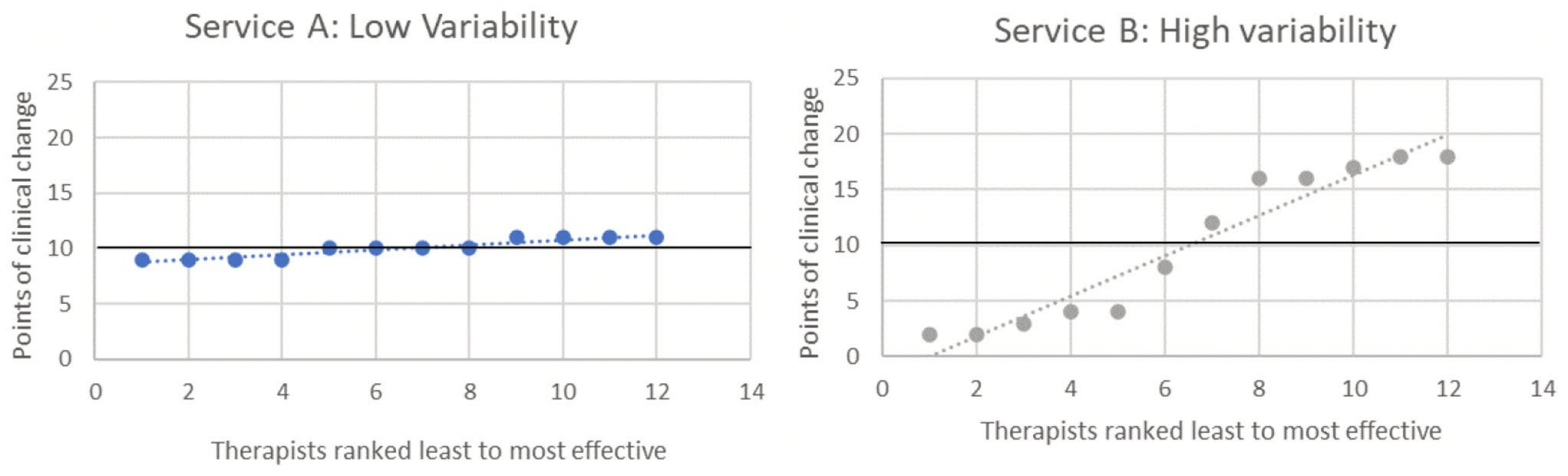
Graph Simulating Services with the Same Group Mean with Low and High Variability

In routine services, measurement of patient outcomes through aggregated group means at a service level can mask considerable variability, and therefore inconsistency, of outcomes being demonstrated between therapists, and hence patients. Figure 1 displays two hypothetical services with identical average patient change of 10 points but with Service A showing minimal therapist variability and Service B showing extensive variability.

The tendency to focus on the mean outcome score at the expense of outcome variability has resulted in limited efforts towards addressing equity of patient outcomes. Managers of psychological therapy services, unless they are a specialist service, have minimal control over the variability between patients entering a universal primary service. By contrast, they are likely to have greater influence over the selection and appointment of therapists employed by a service. If a service is aware of the variability between therapists, it may be able to address this issue via training, supervision, or support. Focusing on both improving patient outcomes *and* reducing therapist variability provides the possibility of creating a more effective but also more equitable service for patients.

Delgadillo et al. (2022) addressed this twin issue of effectiveness and greater equitable services via a meta-analysis of six randomized controlled trials comparing the use, or not, of progress monitoring and their impact on therapist effects. The hypothesis was that progress monitoring, otherwise termed routine outcome monitoring (ROM), would reduce therapist effects by making therapists’ outcomes more homogeneous. Results showed a reduction in therapist effects from 0.011 to 0.009 (i.e., from 1.1 to 0.9%), a result that was statistically significant and presented as an 18.2% drop. However, as noted earlier, investigating therapist effects in trials is questionable, added to which the actual size of the reduction is very small given the context of a trial providing the optimum environment for obtaining the maximum effect under investigation. As ever, the crucial question remains whether effects from trials generalize to routine practice. Hence, the study does not answer the question of whether reducing therapist effects – that is, delivering greater equity – can be achieved in routine practice.

Regardless of research methodologies, research efforts have focused on implementing generic activities such as routine outcome monitoring and feedback (see Barkham et al., 2023), as well as supervision being a standard intervention, and more recently a focus on deliberate practice (Ericsson et al., 1993). One notable effort at implementing these approaches has been the report on the Calgary Counselling Centre in which the outcomes of 153 therapists over 7 years were monitored in response to the implementation of feedback and deliberate practice (Goldberg et al., 2016). Results showed an average improvement (*d*) over time of 0.035 units, which was not attributable to the impact of more recently appointed personnel. This naturalistic study is informative but is difficult to ascertain the specific impacts of the different methods used to improve outcomes and, crucially, focused on improving effectiveness rather than effectiveness and equity.

Given these collective findings, and by adopting a proof of concept design within a practice-based research paradigm, we carried out a case study of a service that was deemed to be failing and requiring a program of external support. The single service was based within the English Talking Therapies (for anxiety and depression) program, previously known as Improving Access to Psychological Therapies (IAPT; Clark, 2018). In response, the service devised a plan of action to deliver both a more effective and equitable service. To that end, organizational and individually-targeted interventions were designed and delivered consistent with components of an informal learning health system. A key component of the Talking Therapies program is the nationally mandated administration of routine outcome data at every patient-attended therapy session. Such a feature is a defining hallmark of a learning health system where the generation of evidence is viewed as “a by-product of care delivery and [the] application of that evidence to support continuous improvement, evidence-based care delivery, and population management” (Guise et al., 2018, p. 2237). In effect, it is where “evidence is both generated and applied as a natural product of the care process” (Ramsberg & Platt, 2017; see also Green et al., 2012).

In addition to the clinical service having available data at every session and a culture and commitment to learn from their own data, there was also a key staff member who was both a manager and in-house data analyst, as well as funding to support the agenda to transform the clinical service. Adopting a learning health system has been identified as a key feature of adapting to changing mental health policies and conditions (O’Sullivan, 1999). Within this context, our aim was to document the sequential impact of, first, a management-led focus on patient outcomes followed by an individually-targeted program focused on reducing practitioner (i.e., therapist) variability as a means for transforming the effectiveness and equity of a clinical service back to a healthy status.

## Method

### Clinical Setting

The setting for the current study was a large Talking Therapies for Anxiety and Depression service, part of the English National Health Service (NHS) Talking Therapies for Anxiety and Depression program, previously known as the Improving Access to Psychological Therapies (IAPT) program (Clark, 2018). The Talking Therapies initiative introduced access to NICE-recommended psychological therapies for people with common mental health problems in primary care settings across England (Clark, 2018). These services are accessible via self-referral, as well as professional referral, and therapies are offered using a stepped care approach, with Low Intensity Cognitive Behavioral Therapy interventions provided at Step 2, and a range of NICE-recommended high intensity therapies at Step 3: Cognitive Behavioral Therapy (CBT); Person-Centered Experiential Counseling for Depression (PCE-CfD); Interpersonal Psychotherapy (IPT); Eye Movement Desensitisation and Reprocessing (EMDR); Couples Counseling for Depression (CCfD); Dynamic Interpersonal Therapy (DIT) (NHSE & NCCMH, 2021). Multiple session-by-session patient outcome measures are used routinely within Talking Therapy services, making them the most highly measured mental health services in the NHS (Gyani et al., 2013). The current study focuses on high intensity (i.e., Step 3) therapy provision.

### An Underperforming Service: Case Study

The service comprised a large psychological therapies service in a part of England, UK that includes large rural areas and a small number of urban centres with a population of just over 1 million in an area of approximately 5,500 km. Based on the Office of National Statistics (ONS) 2021 census data, 24% of the population was aged 65yrs or over and 95% of the population described themselves as an ethnic group of White, compared to 81% of the population of England. The service operated a ‘hub and spoke’ delivery model, with five main clinic and administration sites but with clinical staff also providing services in multiple smaller, shared venues such as General Practitioner (GP) surgeries, community centers, colleges, and supermarkets.

**Fig. 2:**
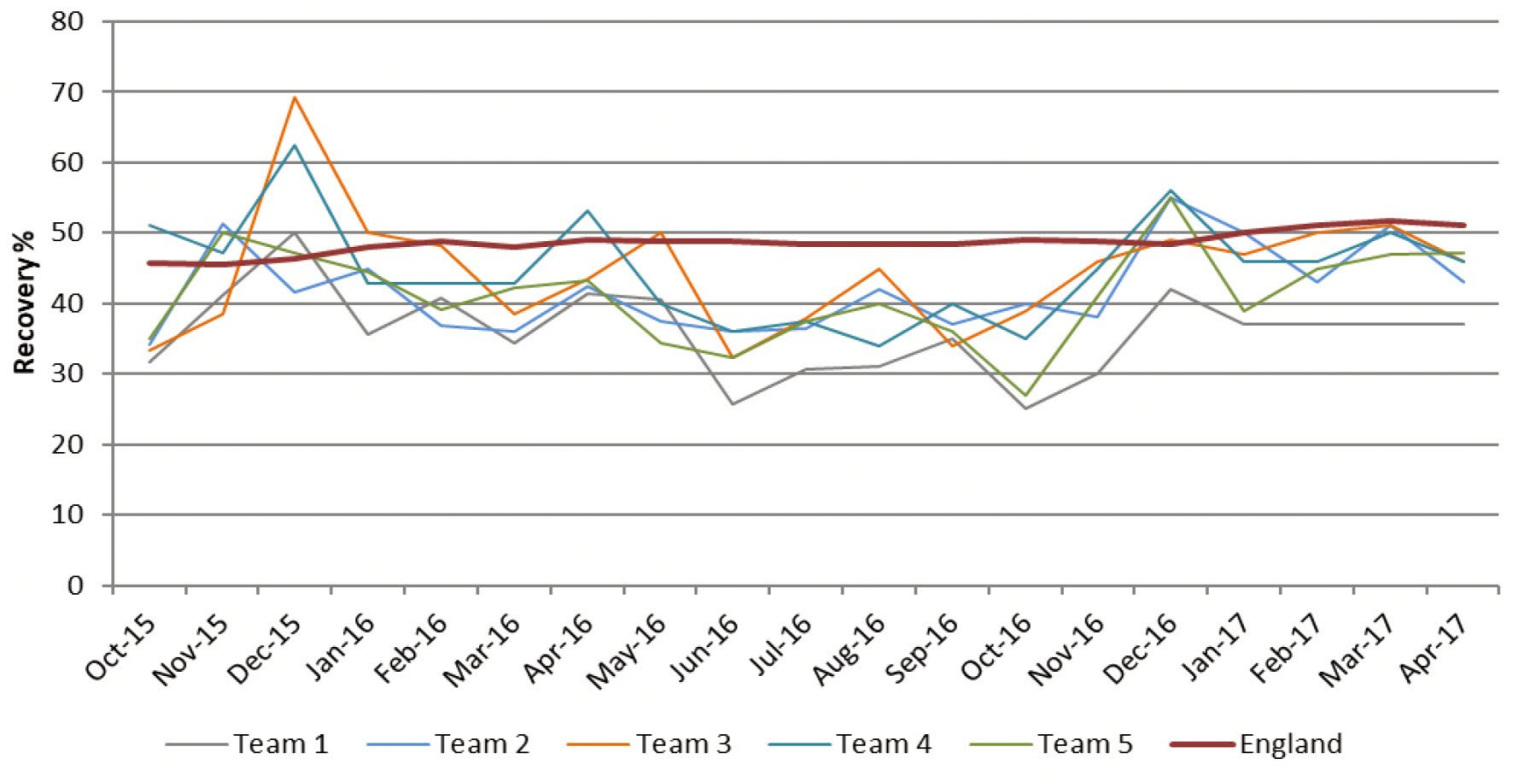
Service Recovery Rates Oct 2015-Apr 2017 (Data source Internal Service Data)

The service comprised five clinical teams and was evidencing lower overall clinical performance than required by national standards. The clinical recovery outcomes for the services’ five teams, with England as a comparator, are shown in Fig. 2 in which the expectation is that services deliver recovery rates of 50 per cent. The data in Fig. 2 shows the below average outcomes across all five teams within the service, particularly during the period from February to November 2016. This poor performance resulted in the service being audited by National Health Service Improvement (NHSI) in January 2017 with requirements to improve clinical outcomes. The audit undertaken by the NHSI Intensive Support Team involved interviews and focus groups with staff and managers across the service as well as an in-depth analysis of the service performance data. Conclusions were provided in a report that was shared with the service, local commissioners, and NHS England (NHSE). A number of recommendations were made within the report and the service was expected to use these recommendations to develop and implement a recovery program with the end result of improving clinical outcomes. Progress was monitored by monthly progress meetings with service senior management, lead commissioners and a representative from either NHSI or NHSE for 12 months following the audit, after which it was deemed that the service was progressing sufficiently to return to standard assurance and monitoring processes.

### Service Recovery Program

The recovery plan comprised three sequential and distinct 18-month phases comprising analyses of pre-existing data as well as the collection and analyses of ongoing data. The three phases were as follows. First, a retrospective baseline phase of patient outcomes was extracted from archived data. Second, a management-led ‘top-down’ intervention phase aimed at improving patient outcomes (i.e., effectiveness target). And third, a bespoke ‘bottom-up’ phase aimed at reducing practitioner variability without compromising patient outcomes (i.e., equity target). This longitudinal design, with each phase comprising 18 months, allowed for an evaluation of the two intervention phases, acknowledging that the bottom-up phase (equity) would be additive to the top-down phase (effectiveness). Importantly, however, the focus on equity was not present in the top-down phase.

The dataset used for the evaluation was extracted from the clinical patient management system IAPTUS (www.iaptus.co.uk), which is a user friendly, cloud based and customizable electronic patient record (EPR) built to support psychological therapies and used in approximately two thirds of Talking Therapies services across England. This system records patient demographic information as well as session clinical notes, clinical outcome measure scores and other relevant metrics (including suicide risk level, use of psychotropic medications, number of previous therapy episodes, satisfaction feedback).

In the context of this recovery plan, and in order to reduce the impact of possible confounding variables, in particular turnover of practitioners, in this report we primarily focus on the cohort of 35 practitioners, comprising CBT therapists and Person-Centered/Experiential counselors, who were constant throughout the period of the three phases of the study. These 35 practitioners represented approximately 28% of the total number of practitioners (126) across the three phases of the study: Phase 1, 35/81 = 43.2%; Phase 2, 35/80 = 43.7%; and Phase 3, 35/74 = 47.3%. However, as a validity check in the context of the total service, we also present data on the whole sample of practitioners employed by the service during the duration of the study.

**Fig. 3:**
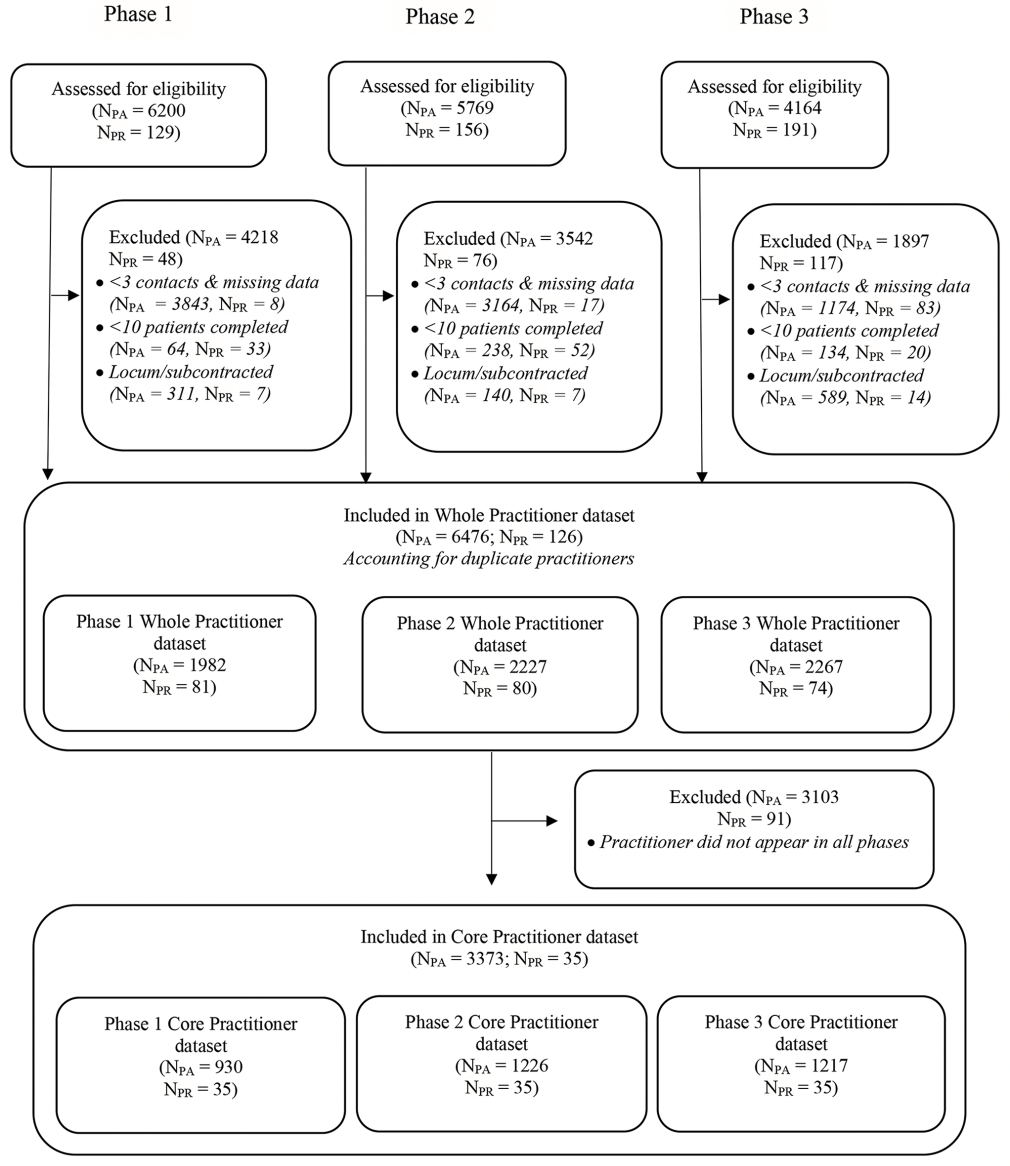
Whole and Core Practitioner Datasets from Full Dataset CONSORT diagram

Figure 3 represents a dataflow diagram from the patients and practitioners eligible for the study, through to the full dataset comprising 6476 patients and 126 practitioners from which the primary study sample was derived. For the primary study sample, a balance was struck between creating as large a sample as possible of ‘core’ practitioners who had data in each phase, while ensuring each saw an adequate number of patients at each phase to produce reliable model estimates. We set a criterion of a minimum 10 patients per practitioner at *each* of the three phases, thereby yielding a minimum of 30 patients per practitioner across the duration of the study and yielding a total sample of 35 ‘core’ therapists and 3373 patients. For each phase the patient sample sizes were 930, 1226 and 1217 respectively. Therefore Phases 2 and 3 met Schiefele et al.’s (2017) recommendation of a total patient sample of 1200, as a product of the number of practitioners and their individual patients Although the sample size in the baseline phase fell short of recommendations we reasoned that this shortfall was offset by the benefits of considering effects for the same practitioners across the three phases. A sensitivity analysis of practitioner effects was conducted on an expanded sample, which included therapists who had at least one patient in each phase (N_PR_=53, (1) N_PA_= 1104; (2) N_PA_=1472; (3) N_PA_ =1412). Accordingly, this design enabled direct comparisons of practitioner variability and patient outcomes of the same practitioners across the three phases. In addition, the whole samples of patients and practitioners in each phase ([1] N_PR_ = 81, N_PA_ = 1982; [2] N_PR_ = 80, N_PA_ = 2227; [3] N_PR_ = 74, N_PA_ = 2267) were analysed as a service-wide validation analysis.

#### Ethical Approval

for Phase 1 and 2 of the overall study was granted by the East of England branch of the Health Research Authority (Ref: 17/EE/0251) and for Phase 3 by Health Research Authority and Health and Care Research Wales (Ref: 18/NS/0104).

### Practitioners and Patients

#### Practitioners

Our primary focus was on the cohort of 35 practitioners who had treated a minimum of 10 patient episodes in each phase while excluding any practitioners not appearing in all three phases (see Fig. 3). Basic practitioner demographics are presented in Table 1 for the core therapist sample and also, for comparative purposes, the whole practitioner sample (*n* = 126). Of the 35 core practitioners in the datasets, 68.6% were female and 63% of the sample were CBT therapists. Six of the practitioners (CBT only) were in training during part of Phase 1, comprising 17% of the total practitioners in the sample. In the whole practitioner sample, a higher proportion of CBT was accessed compared to counseling, and to a larger degree in Phase 2 and 3 datasets than for the core practitioners. Proportionally, for counseling there were more male than female practitioners in the core compared with the whole sample, whereas for CBT the proportions were reversed. In addition, across the phases, the distribution of the two professions were more even in the core samples than in the whole samples.

**Table 1:** Basic demographics of core and whole sample practitioners

Study Phase	Practitioner Sample	*N*	CBT (%)	Counselor (%)	Male (%)	Female (%)	Trainee (%)
1, 2, & 3	Core	35	22 (63)	13 (37)	11 (31.4)	24 (68.6)	6 (17)
1	Whole	81	50 (62)	31 (38)	25	75	13 (16)
2	Whole	80	55 (69)	25 (31)	24	76	12 (15)
3	Whole	74	57 (77)	17 (23)	22	78	13 (17)

All qualified CBT practitioners were trained to post-graduate diploma level and accredited by the British Association of Behavioural and Cognitive Psychotherapy. Counselors were accredited by either the British Association for Counselling and Psychotherapy or UK Council for Psychotherapy. Some clinicians also had core professions as mental health nurses, occupational therapists, social workers, or probation officers.

### Patients

Several exclusions were applied to the patient dataset. First, patients with less than three contacts were not included. This was due to an extended assessment model operating within the service whereby it was not possible to differentiate between a two-session assessment and a single assessment and single treatment episode. Defining a therapy treatment as 3 sessions minimum allowed for an assessment and two treatment sessions to constitute a therapy episode. In addition, we excluded patients whose therapy started or ended outside of the 18-month data period for each phase, patients with missing data on key outcome measures, or patients treated by locum or subcontracted practitioners. Descriptive information on the samples of patients are presented in Table 2, which shows more patients were retained in Phase 2 and Phase 3 samples, and a slightly higher proportion of patients received therapy from a CBT practitioner compared to counseling interventions in each phase, with Phase 1 being most evenly split between these two treatment groups. Between 31% and 34% of patients were male across the phases, and the average age ranged from 41 to 46 years. The split of gender of patients remained consistent, with more female patients being treated. The average age of patients ranged from 39 to 44, which was slightly less than the core practitioner sample, although the pattern across the three phases were similar (see Table 2).

**Table 2:** Features of patients in Core and whole practitioner samples

Study Phase	Practitioner Sample	N	Treating Practitioner		Patient Gender		Patient Age (years)	
			Therapist (%)	Counselor (%)	% Male	% Female	Mean (SD)	Range
1	Core	930	471 (50.6)	459 (49.4)	34.1	65.9	45.8 (15.25)	17–94
	Whole	1982	1017 (51.3)	965 (48.7)	35.1	64.9	44.3 (15.80)	17–94
2	Core	1226	691 (56.4)	535 (43.6)	30.8	69.2	41.5 (15.93)	16–94
	Whole	2227	1416 (63.6)	811 (36.4)	33.0	67.0	39.7 (15.96)	16–94
3	Core	1217	650 (53.4)	567 (46.6)	33.2	66.8	40.7 (15.69)	16–90
	Whole	2267	1583 (69.8)	684 (30.2)	32.0	68.0	38.9 (15.31)	16–90

### Phase Interventions

#### Phase 1

Phase 1 represented the service during a period of ‘*service as usual’* and functioned as a baseline for the following two phases of service improvements. It provided the baseline practitioner effects from retrospective data extraction and analysis, with no significant changes occurring within the service delivery. During Phase 1 the service was experiencing lower overall clinical performance than desired.

#### Phase 2

Phase 2 was the implementation of a program of changes agreed by the service management team, as recommended following a diagnostic analysis by a National Health Service Improvement (NHSI) Intensive Support Team. This program of change involved adjustments across all elements of the service and was a ‘top-down’ process with minimal staff consultation, thus termed here as the *Directive Intervention* Phase. The changes were consistent with greater compliance with the IAPT (now Talking Therapies) supervision model (Clark, 2018). This comprised increased, regular, outcome-focused case management and clinical supervision for all therapy staff, from 2 h per month to weekly 1:1 supervision with the aim of providing robust clinical oversight and assurance as to the quality of the treatment being offered. Also, the creation of a dedicated supervisor team with the aim of increasing the consistency and quality within supervision across the Step 3 part of the service.

Key actions were as follows: The implementation of recovery-focused weekly individual case supervision; the development of a dedicated supervisor team to improve the consistency and quality of supervision; the design and implementation of robust assurance processes within supervision related to clinical decision making and treatment fidelity comprising developing treatment package guidance to improve the fidelity to NICE guidance when offering treatment options, to be used in supervision; and implementing regular audits of supervision spreadsheets and treatment adherence.

Improvements to supervision comprised implementing a specific group of Step 3 supervisors formally recruited from the practitioner cohort within the service, with their primary focus being the delivery and development of clinical case supervision across Step 3, including counseling. These supervisors had reduced caseloads (0.2wte of clinical work, 0.8wte of supervision tasks) allowing them to deliver supervision to a larger group of practitioners, thus increasing the chances of improved consistency in supervision and clinical decision making across the service. This group of supervisors was line managed by one lead practitioner who provided support and oversight of the supervision provided by this group. Supervision was increased to weekly 1-hour one-to-one sessions, utilising caseload spreadsheets which provided a summary of the practitioner’s caseload including key clinical information such as: recent clinical outcome scores, problem descriptor, number of attended sessions to date, and date of last attended session. It was mandated that clinical treatment decisions, such as the intervention offered following assessment and the decision to step up/down or discharge a patient, must be discussed in supervision. This ensured greater consistency across the service and assurance that clinical decisions were based on evidence-based practice and NICE guidance. Peer supervision groups ceased, but a monthly clinical team meeting was introduced in each locality area, where Step 3 teams focused on clinically related (non-operational) issues or service changes/updates.

In making these rapid changes, the service aimed to improve the overall clinical outcomes of the service as well as reduce waiting times to a sustainable level. Key interventions in this phase comprised: ensuring full assessments were provided for all patients prior to entering treatment; increased levels of clinical supervision provided by a dedicated supervisor team; other operational adjustments such as waiting list initiatives and demand and capacity planning. Phase 2 allowed a control for the impact that any service intervention may have on overall outcomes but devoid of any focus on practitioner variability, allowing the intervention in Phase 3 to be evaluated against the sequential periods of no intervention (Phase 1) and service-level intervention (Phase 2).

#### Phase 3

*Phase 3* intervention package, in contrast to Phase 2, was explicitly aimed to be carried out in collaboration with therapy staff, hence termed here the *Collaborative Intervention* Phase. The management team during this period were actively incorporating the concept of ‘collective leadership’ (West et al., 2017) into their management practice. Training and self-reflection sessions for managers were introduced throughout this phase with a focus on compassion and authenticity (West et al., 2014) to facilitate this collaborative approach. The main components of the intervention package, developed with therapy staff, comprised the introduction of a ‘deliberate practice-lite’ (DP-lite) package and a series of staff wellbeing events. The DP-lite package was developed based on existing deliberate practice literature (Chow et al., 2015; Ericsson & Lehmann, 1996; Ericsson & Pool, 2016; Goldberg et al., 2016a), adapted to the context of a high volume, cost-constrained service. All clinicians and supervisors were introduced to the concept and activities of ‘deliberate practice’ for psychotherapists (Rousmaniere, 2017) via brief training sessions at the beginning of this phase. Following this, monthly 90-minute deliberate practice peer groups were formed where practitioners practiced micro-skills identified individually for each practitioner within their individual clinical supervision. Four wellbeing events were identified and arranged by the staff group, aimed to increase resilience and self-care.

The pragmatic deliberate practice-lite package comprised two key elements:

1. *Monthly 1–1.5 h deliberate/reflective practice peer groups.* Practitioners were invited to form small peer groups of 3–5 people. Mixed modality groups were encouraged but not mandated. These groups were specifically for practitioners to practice micro-skills within a group setting, benefitting from feedback from the group in a way that was most supportive to the practitioner.
2. *Identification of individualized goals and micro skills within individual supervision.* Supervisors were provided with an additional information/training session on the principles of DP, with a focus on the identification of micro-skills. They were encouraged to support their supervisees to identify particular micro-skills that they and the supervisee felt would benefit from further development. These micro-skills would then become the focus of DP-lite peer group sessions over the coming weeks/months, until the supervisee felt ready to move on to a new skill. Utilization of feedback from listening to therapy tapes (a pre-existing routine, though infrequent, activity in one-to-one supervision in the service) and ROMs was encouraged within the training and at monthly supervisor meetings held within the service.

To support the implementation of the above elements of the package, four additional elements were included across the 18-month period. First, *initial 2 h DP training session for each team of practitioners at the point of implementation*. This session was delivered by the primary researcher and other members of the research team. The session included a summary of the literature underpinning the development of the package, and more detailed information about how DP can be applied in psychological therapy practice. Clips of a video interview with Tony Rousmaniere were used, including examples of DP in action (Psychotherapy Expert Talks, 2017). Second, *2 h follow up review and training session for each team 4–6 months following implementation.* Each team was visited by the primary researcher or embedded co-researcher for a follow-up session between 4 and 6 months following the initial training session. The session focussed on obtaining feedback from practitioners about whether they had used the ideas of DP, obstacles or reasons for not using DP, examples of using DP, and reflections or feedback on DP and the new groups. The session also included a refresh of the rationale and purpose of DP. Third, *2 h top-up training session via webinar for the whole service 12 months following initial implementation*. A webinar was conducted by the primary researcher and embedded co-researcher 12 months following the initial implementation of the DP-lite/reflective groups. The webinar recapped the rationale and purpose of DP and primarily focussed on the practical application of DP in action. The researchers provided examples of the application of DP in their own practice as well as utilizing feedback from the attendees to troubleshoot and share best practice. Fourth, *research attendance at supervisor meetings every 3 months.* The primary or co-researcher would attend a pre-existing clinical supervisor meeting approximately every 3 months to specifically discuss the DP-lite package, provide any additional information and answer questions that supervisors may have. This functioned to troubleshoot any difficulties and share learning across the service.

### Psychological Interventions

Consistent with nationally reported data (see NHS Digital, 2022), the two psychological interventions offered to patients mainly comprised Cognitive-Behavioral Therapy (CBT; Beck, 2020) delivered by CBT therapists or Person-Centered Experiential-Counseling for Depression (PCE-CfD; a form of Person-Centered Experiential Therapy; see Barkham et al., 2021; Duffy et al., 2023; Murphy, 2019) delivered by counselors. In addition, the service offered eye movement desensitisation and reprocessing (EMDR; Shapiro, 2017) delivered by CBT therapists, or Interpersonal Psychotherapy (IPT; Markowitz & Weissman, 2004) delivered by counselors. In line with national data, EMDR and IPT interventions accounted for less than 5% of courses of treatment offered to patients. Sessions were not capped and therapy was offered in line with National Institute for Health and Care Excellence (NICE) Guideline recommendations for the relevant diagnosis/presenting problem (NICE, 2009, 2022). All interventions were approved by the NHS Talking Therapies program and therapists and counselors received regular supervision from senior practitioners.

### Outcome Measures

The outcome measures available were those collected routinely by NHS Talking Therapies services nationally, with the primary outcome measure being the pre-post change on the Patient Health Questionnaire-9 (PHQ-9; Kroenke et al., 2001; Spitzer et al., 1999). This is a nine-item measure of depressive symptoms, capturing the primary features of depression included in the Diagnostic and Statistical Manual of Mental Disorders (DSM-IV; American Psychiatric Association, 2013). PHQ-9 is an established measure for depression with good internal consistency (Cronbach α = 0.89), test-retest reliability (intraclass correlation = 0.84), and sensitivity and specificity (see Kroenke et al., 2001 for details).

The outcome metric used within the analysis was the amount of change on the PHQ-9 that a patient demonstrated from first to last Step 3 therapy session, determined by the difference between the first and final PHQ-9 at attended sessions. The reliable improvement definition for PHQ-9 score change (Evans et al., 1998; Jacobson & Truax, 1991) was used as a primary measure of clinical improvement, in line with service definitions, namely a reduction of 6 or more points (McMillan et al., 2010).

Secondary measures mandated in routine practice across the service were used to compare the samples: Generalized Anxiety Disorder-7 (GAD-7; Spitzer et al., 2006), a 7-item measure of symptoms of Generalised Anxiety Disorder used in primary healthcare settings; The Work and Social Adjustment Scale (WSAS; Marks, 1986; Mundt et al., 2002), a 5-item measure of functional impairment in relation to a specified disorder; and the Phobia Scale, (DH, 2011) which was developed for use in Talking Therapies services to provide a measure of specific anxiety, alongside the GAD-7.

### Control Variables

In order to estimate practitioner variability, patient variables available at intake and that were significantly associated with outcome were controlled for in the analysis. Potential patient control variables available were: intake scores on PHQ-9, GAD-7, WSAS, and Phobia score and patient demographics comprising age, gender, and deprivation. For deprivation, the English Index of Multiple Deprivation (IMD) (Department for Communities and Local Government, UK Government, 2015) was used. Practitioner variables available were limited to gender, qualification status, and core therapeutic training type. Continuous variables were added grand-mean centered to aid interpretation (Snijders & Bosker, 2011).

### Statistical Methodology

The primary analysis used multilevel modeling (MLM) to estimate the size of practitioner effect in each study phase (Snijders & Bosker, 2011). For each phase model, statistically significant patient explanatory variables were included first before practitioners were included and improvements in model fit were tested by comparing the change in the − 2*loglikelihood ratios against the chi squared statistic for the additional degree of freedom. In order to test whether the practitioner effects found in each phase were reliably different from each other, Markov Chain Monte Carlo (MCMC) simulation procedures were used to provide a measure of uncertainty around estimates of the practitioner effects (Browne, 2015; Browne & Rasbash, 2009; Snijders & Bosker, 2012). Akin to 95% confidence intervals (CIs), these are termed 95% probability intervals (PrIs) and represent the 2.5 and 97.5 percentile values of the practitioner effect taken from a simulation chain. Patient outcomes were defined as pre-post change on PHQ-9 and these were compared across phases using ANCOVAs.

The practitioner residuals produced by the models were ranked and plotted for each phase with their 95% confidence intervals (CIs) in a ‘caterpillar’ plot. The average practitioner change denoted on the plot allows a visual indication of the extent of practitioner variability. Also, as the practitioner residuals represent the extent to which each practitioner increases or decreases their patients’ PHQ-9 outcomes (pre-post change) from the average practitioner, the PHQ-9 values are, therefore, a practical measure of the impact that each individual practitioner has on their patients’ outcomes relative to other practitioners in the sample.

MLwiN software (version 3.02, Rashbash et al., 2009) was used for the multilevel and MCMC analysis, and IBM SPSS Statistics Version 25.0 (2017) was used for all other analyses in the study. These analyses included a comparison of the main features of the dataset of each phase of the study (e.g., average initial/final scores on each measure, reliable improvement rates, score change on each clinical measure, average number of sessions attended, and percentage of cases completed vs. dropped out/referred on). The main analysis focused on those core practitioners present across the three phases but the same analytic procedures were used for the sensitivity analysis of practitioners with at least one patient in each phase and the validity analysis of all patients and therapists in each phase.

### Selection of Predictor Variables

For each sample, a single level regression model was created which included significant patient variables determined by z-scores. The practitioner level was then added, and the practitioner level variance assessed for significance (z-score) and improvement in model fit assessed using the reduction in the − 2*loglikelihood ratio. Random slopes and interactions were also tested for significance.

Models were produced for each phase within each of the core and whole practitioner samples and were compared, identifying common variables and assessing the effect on model fit of inclusions and exclusions where differences occurred. Variables that were consistently associated with outcome across each model were retained.

Following the identification of common patient predictors, the practitioner level introduced in the models and tested for significance and improvement in model fit as above. Practitioner effects were then calculated for each model. Finally, each model was run using MCMC and 95% PrIs were calculated for each practitioner effect to produce a level of uncertainty around effect estimates in order to make comparisons across phases. For the six final models, model assumptions of normality and homoscedasticity were tested and assessed.

## Results

### Descriptive Data

Differences between initial clinical measures across the three phases of the core practitioner sample were small. Table 3 presents the pre-post scores for the core practitioner sample (and whole sample for comparison). Focusing on the core dataset, there was a significant difference between mean initial WSAS scores between phases, *F*(2,3370) = 21.78, *p* < .001. The difference was significant between Phase 1 and 2 (*t*(2154) = 6.26, *p* < .001), Phase 2 and 3, (*t*(2441) = − 4.46, *p* < .001), and Phase 1 and 3, (*t*(2145) = 2.29, *p* < .05). Initial Phobia scores were not normally distributed and there was a significant increase in mean scores, *K-W*(2) = 10.04, *p* = .007. In pairwise comparisons there was a significant difference between Phase 2 and Phase 3, (*p* = .005), but not between Phase 1 and Phase 2 (*p* = .199) or Phase 1 and Phase 3 (*p* = .837).

**Table 3:** Core and whole practitioner sample – Pre/Post clinical scores

Study Phase	Practitioner Sample	PHQ-9 (SD)			GAD-7 (SD)			WSAS (SD)			Phobia (SD)		
		Pre	Post	Change	Pre	Post	Change	Pre	Post	Change	Pre	Post	Change
1	Core	14.7 (6.06)	8.6 (6.58)	6.1 (6.32)	13.1 (5.23)	7.4 (5.59)	5.6 (5.74)	20.1 (9.19)	14.3 (10.37)	6.2 (9.60)	8.9 (6.57)	6.2 (6.35)	2.7 (5.81)
	Whole	15.0 (6.15)	9.06 (6.81	5.9 (6.39)	13.2 (5.20)	7.9 (5.82)	5.3 (5.80)	20.3 (9.29)	14.3 (10.36)	6.3 (9.62)	9.1 (6.68)	6.5 (6.54)	2.6 (5.97)
2	Core	14.7 (5.77)	8.2 (6.21)	6.6 (6.05)	13.4 (4.95)	7.5 (5.27)	5.9 (5.45)	17.6 (9.22)	12.1 (9.37)	5.7 (8.57)	8.3 (6.40)	5.5 (5.82)	2.9 (5.26)
	Whole	15.0 (5.85)	9.0 (6.60)	6.0 (6.18)	13.5 (4.98)	8.2 (5.59)	5.3 (5.52)	18.4 (9.39)	13.0 (9.73)	5.7 (8.97)	8.9 (6.46)	6.2 (6.02)	2.8 (5.40)
3	Core	15.2 (5.61)	8.6 (6.00)	6.5 (5.86)	13.4 (4.76)	7.7 (5.22)	5.6 (5.39)	19.3 (8.69)	12.9 (9.60)	6.1 (8.23)	9.1 (6.31)	6.1 (5.97)	3.0 (5.20)
	Whole	14.8 (5.85)	8.8 (6.27)	6.0 (6.00)	13.5 (4.87)	8.0 (5.48)	5.5 (5.51)	19.0 (8.84)	13.0 (9.72)	5.8 (8.44)	9.7 (6.38)	6.5 (6.06)	3.2 (5.35)

Average PHQ-9 change increased from 6.1 in Phase 1 to 6.6 in Phase 2 and 6.5 in Phase 3. However, ANCOVA tests on each mean change score found no significant differences in mean change scores on any clinical measure between the three phases. However, Table 4 shows comparisons between the three phases on key outcomes for evaluations of service and practitioner performance, as well as comparisons with the whole practitioner sample, and indicates that there was a significant increase in the PHQ-9 reliable improvement rate, of 6.2% points over the three phases ($\:{X}^{2}$ (2) = 8.74, *p =* .013), increasing from just under 50% in Phase 1, 55% in Phase 2, and 56% in Phase 3 ($\:{X}^{2}$ (1) = 8.06, *p =* .005).

**Table 4:** Core and whole practitioner sample – clinical indicators: Reliable Improvement, Therapy Completion and Number of Sessions

Study Phase	Practitioner Sample	PHQ-9 Reliable Improvement Rate N (%)	Therapy Completion Rate N (%)	Session Numbers (SD)		
				Offered	Attended	Missed
1	Core	463 (49.8)	601 (64.6)	9.0 (4.54)	7.2 (3.73)	1.8 (1.99)
	Whole	974 (49.1)	1226 (61.9)	9.0 (4.73)	7.2 (3.95)	1.8 (2.02)
2	Core	671 (54.7)	960 (78.3)	9.2 (4.90)	7.2 (4.03)	1.9 (1.97)
	Whole	1149 (51.6)	1648 (74.0)	9.1 (4.86)	7.1 (3.94)	2.0 (2.05)
3	Core	681 (56.0)	931 (76.5)	10.4 (5.23)	8.2 (4.26)	2.2 (2.13)
	Whole	1157 (51.0)	1764 (77.8)	11.0 (5.55)	8.6 (4.58)	2.3 (2.25)

Table 4 also shows that therapy completion rate (number of patients who completed therapy rather than those who were stepped up, or discontinued therapy early) increased significantly across the phases ($\:{X}^{2}$ (2) = 58.05, *p* < .001). There was a significant increase in completion rate between Phase 1 and Phase 2 ($\:{X}^{2}$ (1) = 49.53 *p* < .001), from 64.6% of patients to 78.3% of patients completing, and between Phase 1 and Phase 3 ($\:{X}^{2}$ (1) = 36.37, *p* < .001), with 76.5% of patients completing therapy in Phase 3, which corresponded to a small, non-significant decrease in completion rate between Phase 2 and Phase 3. The average number of attended sessions also increased significantly (K-W(2) = 43.85, *p* < .001). Differences were significant between Phase 2 and 3 (*p* = .000, *r* = − .12) and Phase 1 and 3 (*p* < .000, *r* = − .11), but not between Phase 1 and Phase 2 (*p* = 1.0, *r* = .01).

### Practitioner Effect

In order to compare practitioner effects across the three phases, the common patient (case-mix) variables significantly associated with outcome in each phase were identified in a preliminary analysis. A multilevel model for pre-post change in PHQ-9 was developed for each of the three phase samples. Table 5 shows the significant variables in the context of the multilevel models and includes the level 1 and level 2 variances and the practitioner effect in each phase.

**Table 5:** Estimates from multilevel models

MLM Values	Phase 1		Phase 2		Phase 3	
	Value	S.E.	Value	S.E.	Value	S.E.
Intercept: Average practitioner PHQ-9 change	6.13	*0.30*	6.66	*0.26*	6.86	*0.21*
First PHQ-9	0.56	*0.04*	0.59	*0.03*	0.59	*0.03*
First Phobia	-0.14	*0.03*	-0.05	*0.03*	-0.08	*0.03*
First WSAS	-0.06	*0.03*	-0.10	*0.02*	-0.10	*0.02*
Interaction PHQ-9/WSAS	-0.01	*0.00*	-0.01	*0.00*	-0.01	*0.00*
Level 2 (practitioner) variance	1.50	*0.66*	1.32	*0.52*	0.47	*0.29*
Level 1 (patient) variance	29.38	*1.39*	26.77	*1.10*	25.22	*1.04*
Practitioner (Therapist) effect	4.9%		4.7%		1.8%	

The total variance (level 1 plus level 2) reduced across the three phases from 30.88 in Phase 1 to 25.69 in Phase 3, a 16.8% reduction in overall outcome variance. The proportion of variance attributable to the practitioners reduced most, with a 68.7% reduction in practitioner variance in Phase 3 compared to Phase 1. The patient variance reduced by 14.2% over the same period. Therefore, outcomes became more consistent in Phase 3 due to both patient and practitioner factors but, as a proportion of the variance in Phase 1, practitioner factors showed the larger reduction, hence the smaller practitioner effect. Also, the reduction in practitioner variance indicated that in Phase 3 practitioners were more similar to one another in relation to their patient outcomes. In Phase 3, the practitioner level variance of 0.47 was not statistically significant as indicated by the large standard error (0.29).

The practitioner effects in Table 5 indicate that the percentage of the outcome variance due to differences between practitioners reduced from 4.9% in Phase 1 to 4.7% in Phase 2 and a further reduction to 1.8% in Phase 3. In Phases 1 and 2, the practitioner effect was significant at the 0.001 level, as indicated by improvements in model fit when practitioner variability was introduced. For Phase 1 the − 2*loglikelihood ratio of the model reduced by 18.807 which when compared to the chi squared statistic for the additional degrees of freedom (1 for the additional parameter) was statistically significant (*p* < .001). For Phase 2 the reduction was also significant ($\:{X}^{2}$ = 21.906, *p* < .001). The practitioner effect in Phase 3 was not statistically significant at the 0.001 level but was significant at the 0.05 level, as indicated by a small reduction in -2*loglikelihood ratio of 4.44 ($\:{X}^{2}$ = 4.44, *p* = .035). Therefore, although the variance at the practitioner level was not statistically significant, modeling the nested structure still improved model fit in Phase 3 but to a lesser degree than in the other phases.

### Practitioner Residual Plots

**Fig. 4:**
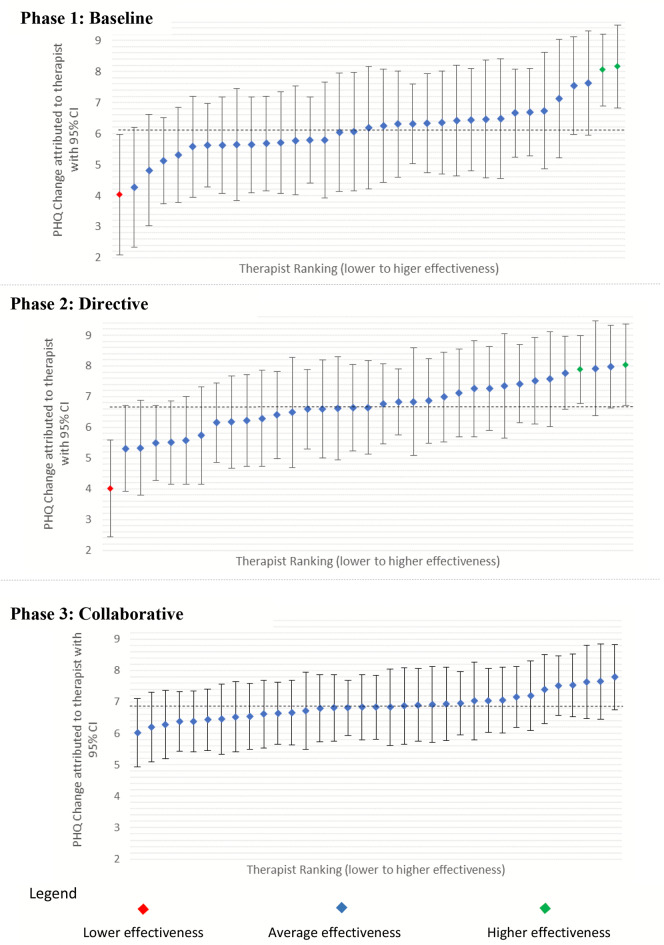
Ranked Practitioners Showing Average PHQ-9 Change for Each Practitioner’s Caseload with 95% Confidence Intervals (CI)

Figure 4 plots practitioner residuals generated by the models with their 95% confidence intervals. The caterpillar plots illustrate the reduction in variability with the points more level in Phase 3 compared with Phase 1 and Phase 2. In Phase 1 and Phase 2 there are two practitioners (to the right, denoted in green) showing significantly higher effectiveness than the average practitioners (denoted in blue) and one practitioner (to the left, denoted in red) showing significantly lower effectiveness than average. The chart for Phase 3 shows the confidence intervals of all practitioners crossing the average residual line, indicating that no practitioner is significantly lower or higher in terms of effectiveness than the average practitioner, illustrating that there is no significant practitioner effect.

In the plots, a residual value of zero has been transformed into PHQ-9 change scores representing the average practitioner pre-post change score (i.e., the model intercept value) for each phase. For example, in Phase 1 the practitioner on the extreme left of the chart has an average change of 4.03 PHQ-9 points, while the practitioner on the extreme right of the chart has an average change of 8.17 points, a difference (i.e., a range) of 4.14 PHQ-9 points. On average, the most effective practitioner obtained more than double the pre-post change for their patients than that of the least effective practitioner. In Phase 2, the difference was 4.02 PHQ-9 points between the least (4.01) and most effective (8.03) practitioners, while in Phase 3, the difference was 1.76 between the least (6.02) and most effective (7.78) practitioner. The ‘levelling out’ of average score change shows more improvement at the lower change end (2 PHQ-9 points), with just a 0.2 of a PHQ-9-point reduction at the upper change end. This indicates that the change has been in uplifting the effectiveness of the therapists at the lower end of effectiveness compared with previous phases.

### MCMC Estimations

**Fig. 5:**
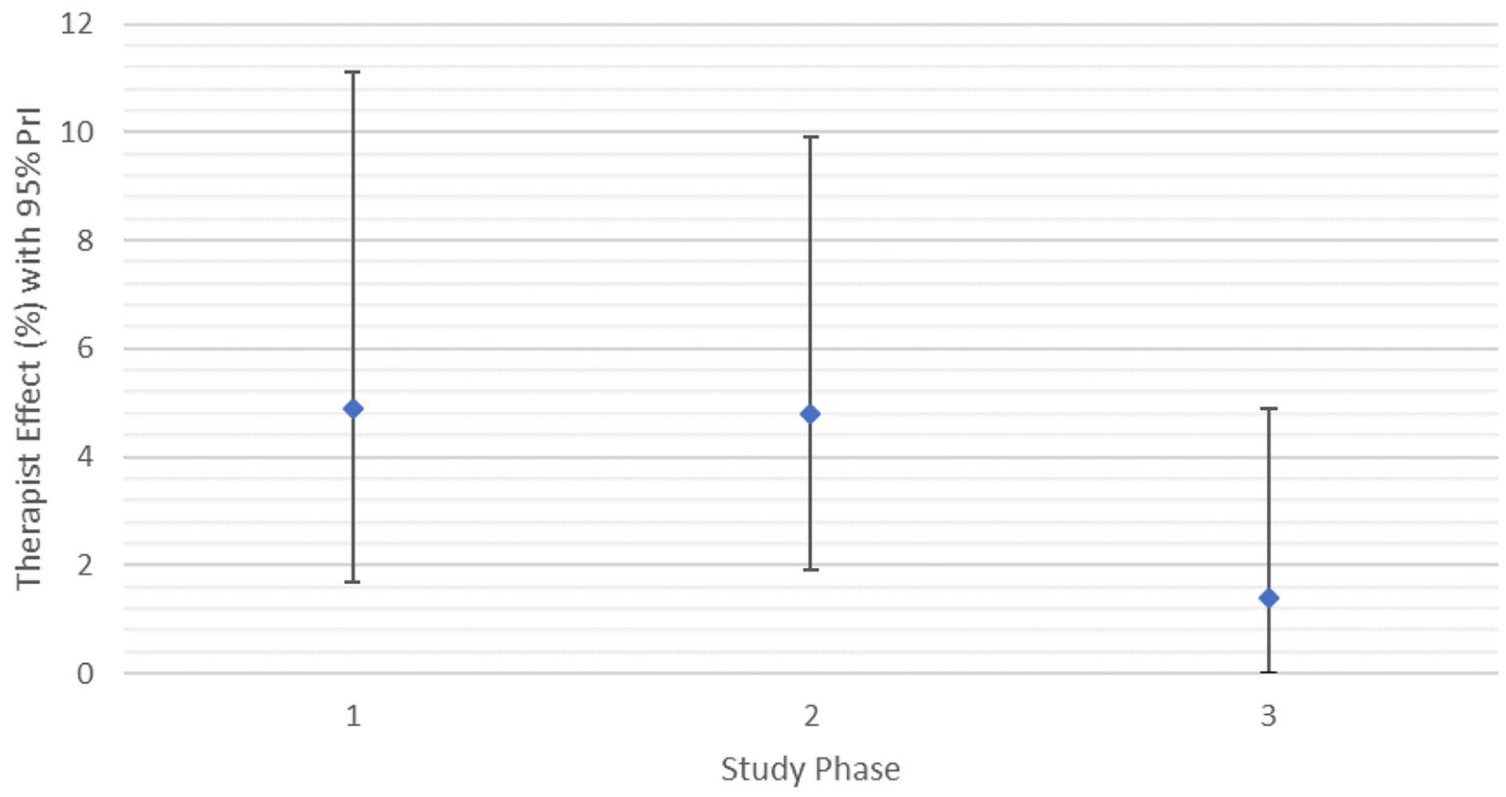
MCMC Therapist Effect (with 95% Probability Intervals) for Each Study Phase

The estimates of practitioner effects may be somewhat unreliable due to the limited number of practitioners included. Therefore, in order to assess the reliability of the effects found, MCMC estimations were derived for each model. These produced 95% Probability Intervals (PrIs), for the practitioner effect in each phase. The estimates and PrIs were plotted as shown in Fig. 5 and demonstrate the reduction in the practitioner effect across phases.

However, as might be expected due to the smaller than optimum number of practitioners, the 95% PrIs for each estimation are wide and overlap, which indicates some unreliability in estimates of effects, although only in Phase 3 was there the probability that the practitioner effect was zero.

### Sensitivity Analysis

To assess the reliability of therapist effects found, a sensitivity analysis was undertaken that included all therapists (*N* = 53) with one or more patients in each phase. The results showed larger therapist effects in phases 2 and 3 than the primary analysis but similarly the smallest therapist effect was found at phase 3. Also, reliable improvement rates increased across phases (see Supplemental Materials A1).

### Validation in the Whole Sample

Finally, we compared the results from the primary analysis with those in the whole sample (i.e. all therapists and their patients in each phase. See also total sample details in Fig. 3). The key points of comparison were: (a) patient improvement rates in Phase 2, and (b) practitioner variability in Phase 3 in addition to patient improvement rates.

Table 6 presents the key data derived from the MLM analysis for both the primary analysis and whole sample analysis. Notable results for the whole sample were that while the practitioner effect increased in Phase 2, it fell to its lowest value in Phase 3 (as with the core practitioners). In terms of patient change, the average practitioner PHQ-9 change through the three phases increased, as it did for core practitioners. And in terms of the separate patient and practitioner levels of variance, both reduced across the three phases of the study. The visual graphic of the whole sample of practitioners with the core practitioners identified within the overall graphic is shown in the Supplemental Materials (see Appendix 1).

**Table 6:** Key Data from the MLM for each study phase of both core and whole therapist samples

Phase	Practitioner sample	Practitioner N	Patient N	Predictive Model – Severity & Functioning (Initial PHQ, WSAS & Phobia w. interaction PHQ/WSAS)				
				Practitioner effect (%)	Average Practitioner PHQ change (SE)	Total variance	Patient level variance (SE)	Practitioner level variance (SE)
1	Core	35	930	4.9	6.13 (0.30)	30.88	29.38 (1.39)	1.50 (0.66)
	Whole	81	1982	4.4	5.97 (0.20)	32.93	31.50 (1.02)	1.44 (0.45)
2	Core	35	1226	4.7	6.66 (0.26)	28.09	26.77 (1.10)	1.32 (0.52)
	Whole	80	2227	6.6	6.03 (0.21)	30.82	28.77 (0.88)	2.05 (0.51)
3	Core	35	1217	1.8	6.86 (0.21)	25.69	25.22 (1.04)	0.47 (0.29)
	Whole	74	2267	3.4	6.19 (0.17)	28.00	27.06 (0.82)	0.94 (0.31)

## Discussion

This longitudinal, practice-based case study aimed to evaluate the recovery of a clinical service from being under intensive support to one of being an effective clinical service. While many metrics could be taken, the two that were the focus of this study were patient change (i.e., effectiveness) and reduced practitioner variability (i.e., an index of equity). Compared with a retrospective 18-month baseline period (no intervention), we monitored effectiveness and equity across two subsequent 18-month phases, the first focusing on improving patient outcomes via an organizational, management-led intervention, and the second by a compassionate individual practitioner focus with the aim of reducing practitioner variability.

In the controlled sample of practitioners (i.e., those constant across the entire three phase time period), we found a marked successive increase in the reliable improvement rates of patient outcomes across the three phases, finalizing at 56%. This trend for patient outcomes was repeated in the whole sample of practitioners but to a lesser extent, although the various rates in the whole sample were very similar to the figure of 51.8% derived from > 6,500 participants and 170 therapists at a large US counseling center (Goldberg et al., 2016). In terms of the extent of reduction in practitioner variability (i.e., therapist effect), the decrease in response to the individualized intervention was notable. Indeed, the practitioner effect fell by 64.4% between Phase 2 (4.7%) and Phase 3 (1.8%) in the context of improved patient outcomes. In short, at Phase 3 patients received an assignment to practitioners that yielded better rates of reliable improvement – a criterion likely to be crucial for services – as well as more equitable outcomes for patients.

Overall, patient outcomes improved and practitioner variability decreased with the exception of Phase 2 for the whole sample of practitioners where the practitioner effect increased before then decreasing in Phase 3. It might be that in a practitioner group that was not constant and responding to a management-led initiative, that the practitioners were responding more as individual practitioners rather than as part of a collective identity, and thereby increasing the variability. There were also differences in the composition of the therapy types delivered by the service across the three phases, however therapy type was not found to be associated with outcome in any analysis. As the study phases were designed to test the impact of a series of changes, or interventions within the context of a single live and dynamic service, it is not possible to attribute causality to the research findings. However, taken together, these results suggest there is sufficient indication that it is possible to reduce practitioner outcome variability whilst maintaining patient outcomes in a stable practitioner sample. Furthermore, practitioner variability may be reduced by low-cost interventions focusing on all or a mixture of the following: micro-skills development/use of deliberate practice, peer-learning, developing a compassionate service culture which aims to build staff confidence, resilience and wellbeing.

While the data indicate a minimal practitioner effect in Phase 3, there is a level of uncertainty around estimates, indicating that they are not reliably different from each other. But, if taking the best estimates of effect, as usually reported in other practitioner effect studies, a reduction in practitioner variability, such as seen in this study, has considerable impact in practice at an individual and service level. Smaller effects have considerable cumulative effects when taken at a population level (Barkham, 2023).

In contrast to the impact of Phase 3 in reducing practitioner variability, Phase 2 did not do so, but the service-level intervention did have a considerable impact on patient completion rates, rising from 65 to 78%, a rate largely maintained in Phase 3. It maybe that Phase 2 constituted a change to the way patients were treated *by the service*, resulting in increased therapy completion (i.e., undertaking robust assessments at point of access ensuring interventions were offered that appeared to be appropriate to patients’ needs and so they continued in therapy). By contrast, in Phase 3, there were targeted changes to the way a patient might be treated *by their practitioner* such that made the delivery of the interventions more uniform and maintained a level of clinical outcome.

### Implications for Practitioners and Services

Although causality is not assumed, given the observations made in this study alongside existing literature, there are a number of activities that warrant adoption by practitioners and services in pursuit of improving both effectiveness and equity within a clinical setting. These activities include learning from peers through deliberate skills practice (Firth et al., 2020; Goldberg et al., 2016a; Miller et al., 2013); reflecting on one’s own practice to identify development areas (Chow et al., 2015; Goldberg et al., 2016a); seeking feedback from others, namely supervisors, peers, and patients through the use of ROMs, to identify potential development areas (Brattland et al., 2018, 2019; Delgadillo, De Jong et al., 2018; Goldberg et al., 2016), In addition to these, taking action to sustain individual resilience and wellbeing (Delgadillo, Saxon et al., 2018; Green et al., 2014; Pereira et al., 2017) may be a factor that practitioners may want to cultivate. The focus on individual case supervision which has traditionally been the key forum for professional learning and development for psychological practitioners, could be supplemented with small peer group opportunities to learn and practice micro-skills with other practitioners, and could include the use of ROMs for the purpose of feedback and learning.

In that context, it is important to define the two interconnected elements of service design and management that may be implicated by the results of the current study. Firstly, the content of the service intervention or model, and secondly the way in which a service intervention or model is implemented and sustained by service leaders and staff. In Phase 2, where improved clinical outcomes (reliable improvement) but higher levels of practitioner variability were observed, the content of the service intervention was primarily focused on the achievement of service targets, delivered in a directive, externally-driven style, with limited staff involvement in decision making or idea generation. In Phase 3, the service model remained consistent with Phase 2, however, additional changes were guided by staff consultation and preferences, with the involvement of staff in the implementation of changes that impacted on them, facilitated by managers committed to a collective leadership approach. If indeed this focus on collaborative working did account for some of the reduction in the practitioner effect, it should be further support for the development of increased reflection and innovation in the realm of change management and health care leadership.

The various components involved in the data collection and subsequent feedback to the service reflect many of the components of a learning health system. Whilst not formally identified as such by the organization, the adoption of significant principles and procedures provides an example whereby the use of embedded and routinely-collected data within a service, (in this case, the PHQ-9), together with the application of sophisticated data analytics (i.e., multilevel modeling) represented by impactful graphics (i.e., caterpillar plots) within the context of a climate of self-reflection, enabled the service to be both a provider and user of its own data for the betterment of both patients and practitioners, the defining hallmark of a learning health system.

### Strengths and Limitations of Study Design and Implementation

The sample size limits any firm conclusions regarding the size of practitioner effects and, in particular, any reliable differences between effects across phases. However, as part of the proof of concept design, a range of comparisons were made between phase models, and in conjunction with clinical outcomes, these were used to assess whether service delivery had improved in Phase 3. The results suggest that it had, but a further multisite randomized study with a sample of over 100 practitioners would be required to make more reliable conclusions about whether practitioner effects had reduced and whether this was a result of the interventions.

A deliberate design feature of the study was to ensure that each phase was of sufficient length to include both an implementation and stabilisation period. On balance and in hindsight, extending each phase to a 2-year time period would have improved the ability to detect any effects more specifically related to the DP-lite aspect of the Collaborative Intervention.

The lack of information about practitioners themselves, such as levels of personal resilience, meant that these variables could not be factored into the study analysis. Having additional measures of key practitioner variables would have further informed the impact of the two intervention phases, or identified other practitioner variables associated with therapist variability. Despite the generalizability of results across the core and whole practitioner samples, it should be noted that the 35 core practitioners were ones who had stayed in the service throughout the 4.5-year study period and may therefore, by definition, be more engaged in change and service improvements. However, conversely, these practitioners had also experienced the most change and service turmoil and one may therefore assume be more likely to be vulnerable to ‘change fatigue’.

In terms of the implementation of the deliberate practice-lite interventions in Phase 3, it should be noted that there were distinct elements of the package that did not meet the general consensus definition for pure deliberate practice (Ericsson & Lehmann, 1996; Miller et al., 2020). First, while individualized learning objectives were included in the training sessions for practitioners and supervisors, they were not monitored during the study other than through self-report feedback at the review sessions. Second, while direct feedback was initiated in Phase 2 and provided as part of supervision through ROMs and therapist outcomes of Talking Therapies recovery and reliable improvement measures continued in Phase 3, this feedback was not directly linked to deliberate practice. Third, while supervisors were senior clinicians within the service, this did not equate to them being expert therapists or experts in psychological therapy nor indeed necessarily having superior patient outcomes compared to their supervisees. Fourth, while the importance of repetition and refinement of skills were included in the training and follow-up sessions, practitioners were provided with deliberate practice time in groups, rather than individually. Finally, none of the therapists, supervisors or DP-lite groups were required to evidence how or if they used DP either within the groups or individually. The purpose of the feedback sessions was to gauge the overall up-take of the ideas within a naturalistic setting, but therapists were not asked for specific information in relation to the amount to which they were using or adhering to the practices as taught.

## Conclusions

The findings from the current study highlight the importance of continued investigations into the impact that service level and therapist level interventions can have on patient outcomes and therapist effects, a significant factor influencing patient outcomes (Wampold & Owen, 2021). They also support efforts to better ensure that the treatment patients receive is equitable as well as supporting the notion of psychological services adopting the principles consistent with being a learning health system in which they become the primary user of the very data they generate in service of better provision for their patients.

## Electronic Supplementary Material

Below is the link to the electronic supplementary material.

Supplementary Material 1
