# Supplementary material for: Transforming the Effectiveness and Equity of a Psychological Therapy Service: A Case Study in the English NHS Talking Therapies Program
Source: Adm Policy Ment Health. 2024 Aug 17;51(6):970–87. doi: 10.1007/s10488-024-01403-0 (PMC11489297; doi:10.1007/s10488-024-01403-0)
Supplement: Supplementary file 1 — Supplementary Material 1 [file 10488_2024_1403_MOESM1_ESM.docx]

**Appendix 1: Supplementary Materials**

**Expanded Core Therapists Sample**

In order to assess the reliability of effects and the representativeness of our main sample, a sensitivity analysis which included all therapists who had at least one patient (N_PR_ = 53) in each phase (N_PA_ =1104, N_PA_ =1472 and N_PA_ =1412 respectively) was also conducted. This aimed to provide an assessment of the reliability of the therapist effect phenomenon observed in the main analysis, as increasing numbers at the therapist level will increase the reliability of the level 2 variance findings, although potentially at the cost of maintaining the reliability of the estimates of the variables in the model. A MLM was calculated for the new expanded core therapist dataset for each phase.

Table A1 presents the findings alongside the therapist effects from the original Core practitioner sample for comparison and shows a reduction in therapist effect from Phase 1 to Phase 3, albeit a smaller reduction than in the original analysis, from 4.3% in Phase 1 to 3.2% in Phase 3. In contrast to the original analysis, an increase in practitioner variance occurred in Phase 2, increasing from 4.3% (Phase 1) to 5.9% (Phase 2) of the total variance residing at the therapist level. Accounting for the increase in therapist effect in Phase 2 in this sample compared to the original, and the larger therapist effect in Phase 3, shows that the reduction in therapist effect from Phase 2 to Phase 3 is in fact similar, with 2.9% points difference in the original dataset, and 2.7% points difference in the expanded sample. Indices of PHQ-9 reliable change were smaller than in the original sample, with an increase from 49.6% in Phase 1 to 53.3% in Phase 3, which was non-significant ($X^{2}$ (2) = 3.61, *p =*.164). These models also demonstrate a consistent PHQ change across the three phases.

Table A1. *Values from Sensitivity Test Multilevel Models with Original Sample Therapist Effects Estimates and Reliable Improvement Rates Shown*

| MLM Values | Phase 1 | | | Phase 2 | | | Phase 3 | | |  |
| --- | --- | --- | --- | --- | --- | --- | --- | --- | --- | --- |
|  | Value | *S.E.* | *p* | Value | *S.E.* | *p* | Value | *S.E.* | *p* | |
| Average therapist PHQ-9 Change (Intercept) | 6.15 | *0.26* | *0.00* | 6.39 | *0.25* | *0.00* | 6.44 | *0.22* | *0.00* | |
| First PHQ-9 | 0.55 | *0.03* | *0.00* | 0.57 | *0.03* | *0.00* | 0.59 | *0.03* | *0.00* | |
| First Phobia | -0.12 | *0.03* | *0.00* | -0.04 | *0.03* | *0.08* | -0.09 | *0.03* | *0.00* | |
| First WSAS | -0.08 | *0.02* | *0.00* | -0.09 | *0.02* | *0.00* | -0.10 | *0.02* | *0.00* | |
| Interaction PHQ-9 / WSAS | -0.01 | *0.00* | *0.01* | -0.00 | *0.00* | *0.03* | -0.01 | *0.00* | *0.00* | |
| Level 2 (therapist) Variance | 1.38 | *0.59* |  | 1.75 | *0.57* |  | 0.88 | *0.38* |  | |
| Level 1 (patient) Variance | 30.71 | *1.33* |  | 28.13 | *1.05* |  | 26.45 | *1.01* |  | |
| Therapist Effect |  |  |  |  |  |  |  |  |  | |
| >=3 Dataset^a^ | 4.3% |  |  | 5.9% |  |  | 3.2% |  |  | |
| >=30 Dataset^b^ | 4.9% |  |  | 4.7% |  |  | 1.8% |  |  | |
| PHQ-9 Reliable Improvement | | | | | | | | | | |
| >=3 Dataset^a^ | 49.8% |  |  | 54.7% |  |  | 56% |  |  | |
| >=30 Dataset^b^ | 49.6% |  |  | 52.6% |  |  | 53.3% |  |  | |

^a^ >=3 Dataset: Therapists with minimum one patient per phase included; ^b^ >=30 Dataset: Therapists with a minimum of 10 patients per phase included

***
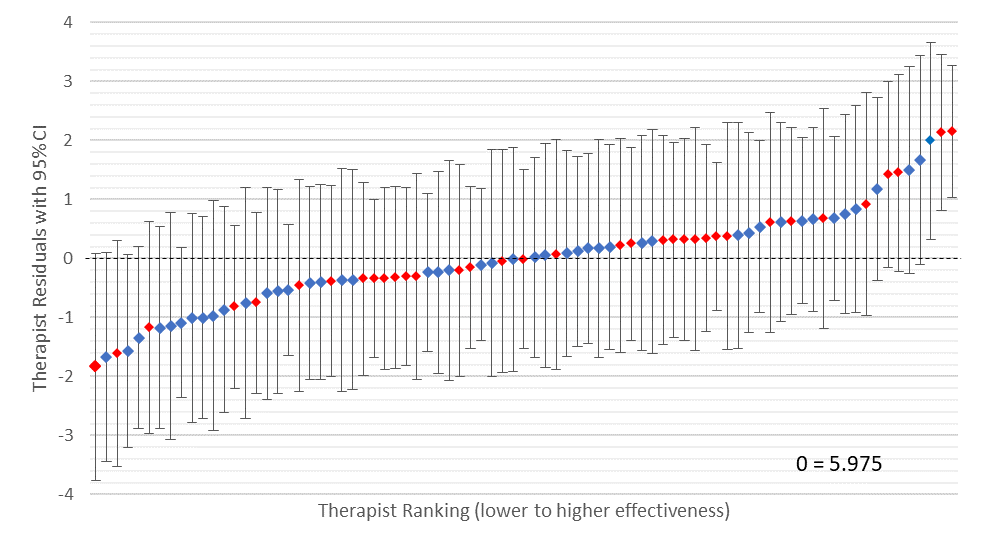

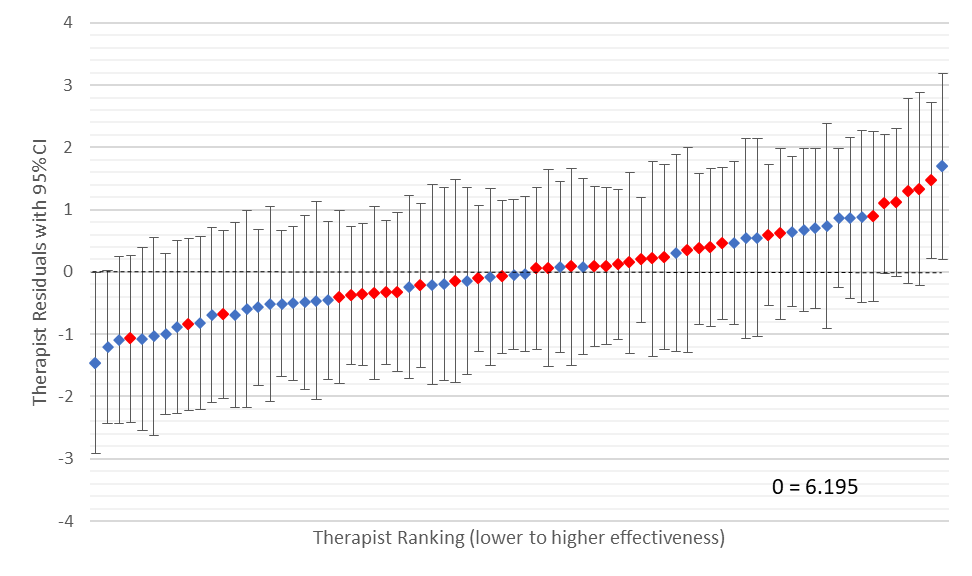
***

In All Therapists sample only Core Therapists

▪

▪

**Phase 1**

***Supplementary Materials: Appendix 1***

*Ranked Therapists Showing All Therapists Residuals with 95% Confidence Intervals (CI) with Core Therapists in red*

**Phase 2**

**Phase 3**

***
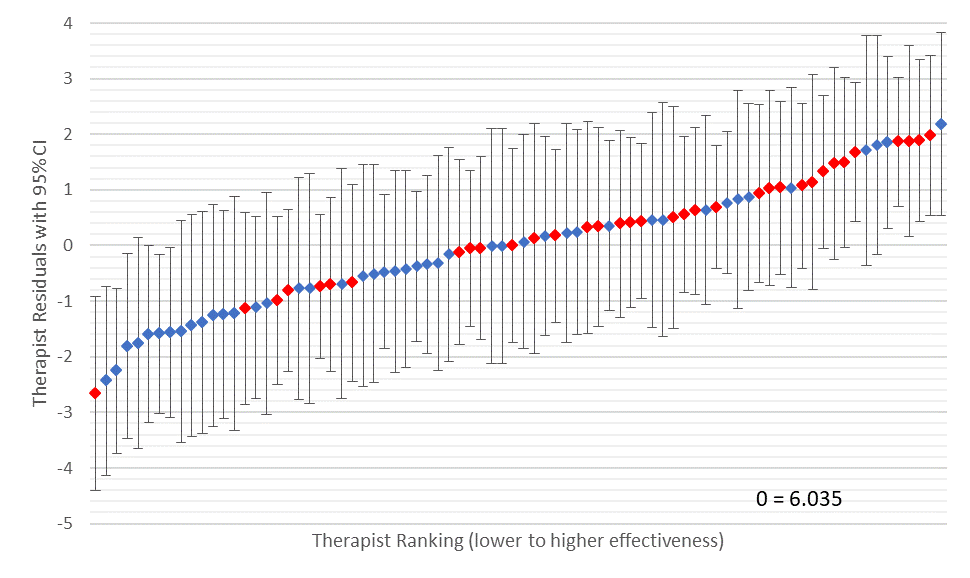
***
